# Implementation of a Manual Vacuum Drainage System for Odontogenic Space Infections: Technical Note

**DOI:** 10.1007/s12663-026-02967-0

**Published:** 2026-03-05

**Authors:** Rafael Alvim Magesty, Carlos José de Paula Silva, Luciana Carvalho Soares Almeida, Endi Lanza Galvão, Saulo Gabriel Moreira Falci, Marcos Luciano Pimenta Pinheiro

**Affiliations:** 1 Universidade Federal dos Vales do Jequitinhonha e Mucuri. Rua da Glória, 187, Centro, Diamantina, 39100-000 Minas Gerais Brazil; 2 Universidade Federal de Minas Gerais. Rua Prof. Moacir Gomes de Freitas, Minas Gerais, 688 - Pampulha, Belo Horizonte, 31270-901 Brazil

**Keywords:** Dentoalveolar Abscess, Drainage Suction, Negative-Pressure Therapies

## Abstract

****Background**:**

Odontogenic space infections typically require surgical incision and drainage for effective source control. While passive drainage remains the standard, manual closed vacuum systems are proposed to enhance exudate evacuation and wound management. This technical note describes the implementation and feasibility of a manual vacuum drainage system in a general hospital setting.

****Methods**:**

The system utilizes a 4.8 mm perforated silicone tube connected to a manual accordion-shaped reservoir. Following surgical debridement, a hermetic seal is created using a 4-0 nylon purse-string suture and reinforced with transparent adhesive film. Negative pressure is generated by manual compression of the reservoir.

****Results**:**

The technique was safe and straightforward to implement. However, operational challenges included frequent loss of the vacuum seal due to patient mobility and drain obstruction by viscous exudate in the absence of continuous irrigation. These factors led to increased nursing maintenance and lower staff acceptability compared to conventional methods.

****Conclusion**:**

Manual closed vacuum drainage is a technically viable and safe alternative for odontogenic infections but is limited by operational constraints in non-intensive care settings. Future refinements, such as integrated irrigation channels, are necessary to optimize clinical performance.

## Technical Note

Odontogenic space infections commonly require surgical incision and drainage to achieve adequate source control [1]. Passive drainage remains the most frequently employed method [2]; however, closed vacuum systems are occasionally considered in an attempt to limit fluid accumulation and improve wound management [3, 4]. This technical note describes the practical implementation of a manual closed vacuum drainage system in the management of odontogenic space infections and reports feasibility-related observations encountered in a general ward setting.

### Technique

**Fig. 1:**
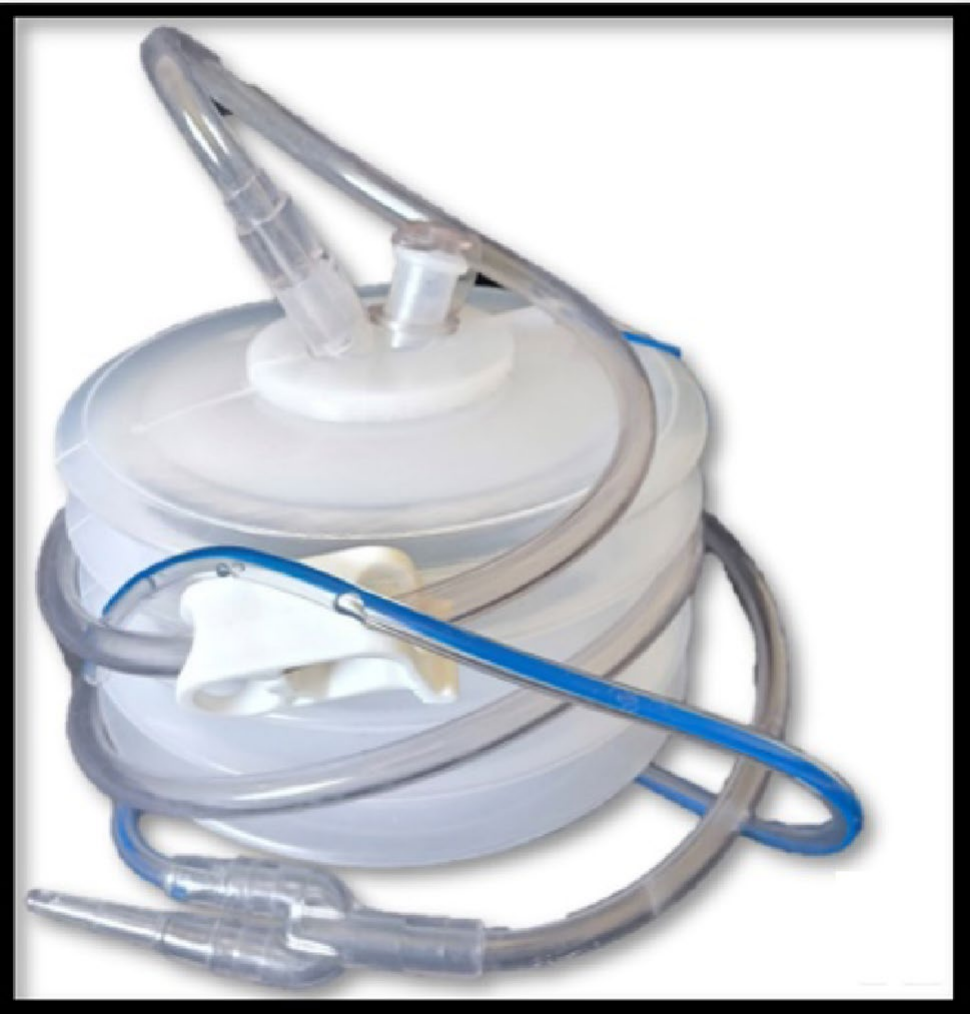
Surgical drain used

**Fig. 2:**
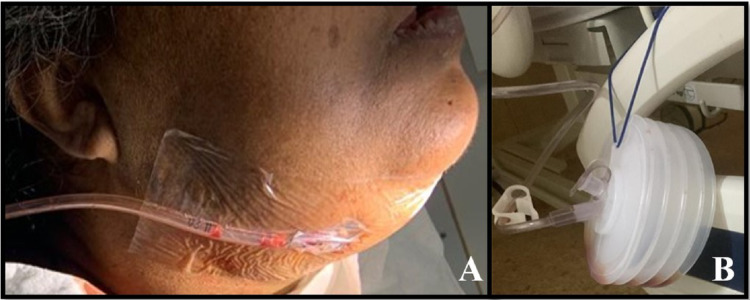
Clinical application of the vacuum drainage system

The proposed system consists of a perforated silicone tube (MedSharp^®^, 4.8 mm) connected to an accordion-shaped manual vacuum reservoir (Fig. 1). After surgical access and evacuation of the infectious focus according to standard institutional protocols, the perforated segment of the drain is positioned within the cavity, ensuring that all fenestrations remain entirely intralesional. To maintain a closed environment, a circumferential 4 − 0 nylon suture is placed at the skin–tube interface, creating a hermetic seal. An additional fixation suture is used to stabilize the tube and prevent displacement. The seal is reinforced with a transparent adhesive film applied circumferentially around the drain entry site (Fig. 2A). The external end of the tube is trimmed and connected to the reservoir hose. Negative pressure is generated by fully compressing the reservoir to expel internal air, followed by closure of the system. During use, the reservoir is kept positioned below the level of the surgical site to facilitate continuous drainage by gravity and vacuum assistance (Fig. 2B). System maintenance is performed at the bedside. When irrigation is required, the adhesive film is temporarily removed, the cavity is flushed with saline solution, and the seal is immediately re-established with a new transparent film to restore negative pressure. The device is typically maintained for up to 72 h and may be removed or replaced if persistent purulent output is observed.

### Drain Management

Maintenance of negative pressure required regular inspection and repeated manual reactivation of the reservoir. As the system lacked a dedicated irrigation channel, intermittent saline irrigation required temporary removal of the adhesive film and brief manipulation of the drain, followed by re-establishment of the seal. Drain patency was assessed clinically based on reservoir expansion, output characteristics, and local wound findings.

### Practical Considerations

Placement of the vacuum drain was technically straightforward for surgeons familiar with extraoral drainage of odontogenic infections. No device-related adverse events or wound complications directly attributable to the drain were observed. However, several operational constraints were consistently encountered. Maintenance of an effective hermetic seal proved challenging in mobile patients, with loss of negative pressure occurring during mandibular movement and routine ward activities. In addition, thick purulent exudate frequently compromised drain patency in the absence of continuous irrigation, necessitating manual intervention. These factors increased nursing workload and reduced overall staff acceptability compared with conventional passive drainage methods.

### Practical Implications

Manual closed vacuum drainage can be technically implemented in odontogenic space infections; however, its performance is limited by mechanical and operational constraints in non-intensive care environments. In its current form, this technique should be regarded as a feasibility-based technical alternative rather than a replacement for established passive drainage systems.

Vacuum drain (MedSharp^®^ 4.8 mm), representing a closed suction system designed for active exudate removal.

## References

[1] Heim N, Warwas FB, Wiedemeyer V, Wilms CT, Reich RH, Martini M (2019) The role of immediate versus secondary removal of the odontogenic focus in treatment of deep head and neck space infections. A retrospective analysis of 248 patients. Clin Oral Investig 23(7):2921–2927. 10.1007/s00784-018-02796-7 30623306 10.1007/s00784-018-02796-7

[2] Bouloux GF, Wallace J, Xue W (2013) Irrigating drains for severe odontogenic infections do not improve outcome. J Oral Maxillofac Surg 71(1):42–46. 10.1016/j.joms.2012.04.043 22726703 10.1016/j.joms.2012.04.043

[3] He D, Qian Y, Zhou L, Qi H, Liu Y (2021) Multifunctional Irrigation-Assisted Vacuum Drainage versus Traditional Drainage in the Treatment of Odontogenic Deep Fascial Infection: A Retrospective Cohort Study. Infect Drug Resist 14:3571–3580. 10.2147/IDR.S326300 34511948 10.2147/IDR.S326300 PMC8421558

[4] Qian Y, He D, Qi H, Zhang T, Fu T, Yu M, Yan Q, Cai Z, Liu Y (2022) Comparative Study of Multifunctional Irrigation-assisted Vacuum Drainage, Vacuum Sealing Drainage and the Penrose Drain in Treating Severe Multi-space Deep Fascial Infection in the Head and Neck. World J Surg 46(12):2973–2983. 10.1007/s00268-022-06758-9 36216895 10.1007/s00268-022-06758-9

